# Do we need repeated CT imaging in uncomplicated blunt renal injuries? Experiences of a high-volume urological trauma centre

**DOI:** 10.1186/s13017-022-00445-9

**Published:** 2022-07-07

**Authors:** Andrea Katharina Lindner, Anna Katharina Luger, Josef Fritz, Johannes Stäblein, Christian Radmayr, Friedrich Aigner, Peter Rehder, Gennadi Tulchiner, Wolfgang Horninger, Renate Pichler

**Affiliations:** 1 Department of Urology, Medical University of Innsbruck, Anichstraße 35, 6020 Innsbruck, Austria; 2 Department of Radiology, Medical University of Innsbruck, Innsbruck, Austria; 3 Department of Medical Statistics, Informatics and Health Economics, Medical University of Innsbruck, Innsbruck, Austria

**Keywords:** Blunt renal trauma, Repeat imaging, Non-operative management, Selected imaging, Selective angioembolization

## Abstract

**Background:**

Current guidelines recommend repeat computed tomography (CT) imaging in high-grade blunt renal injury within 48–96 h, yet diagnostic value and clinical significance remain controversial. The aim of this work was to determine the possible gain of CT re-imaging in uncomplicated patients with blunt renal trauma at 48 h after injury, presenting one of the largest case series.

**Methods:**

A retrospective database of patients admitted to our centre with isolated blunt renal trauma due to sporting injuries was analysed for a period of 20 years (2000–2020). We included only patients who underwent repeat imaging at 48 h after trauma irrespective of AAST renal injury grading (grade 1–5) and initial management. The primary outcome was intervention rates after CT imaging at 48 h in uncomplicated patients versus CT scan at the time of clinical symptoms.

**Results:**

A total of 280 patients (mean age: 37.8 years; 244 (87.1%) male) with repeat CT after 48 h were included. 150 (53.6%) patients were classified as low-grade (grade 1–3) and 130 (46.4%) as high-grade (grade 4–5) trauma. Immediate intervention at trauma was necessary in 59 (21.1%) patients with high-grade injuries: minimally invasive therapy in 48 (81.4%) and open surgery in 11 (18.6%) patients, respectively. In only 16 (5.7%) cases, intervention was performed based on CT re-imaging at 48 h (low-grade vs. high-grade: 3.3% vs. 8.5%; *p* = 0.075). On the contrary, intervention rate due to clinical symptoms was 12.5% (n = 35). Onset of clinical progress was on average (range) 5.3 (1–17) days post trauma. High-grade trauma (odds ratio [OR]_grade 4 vs. grade 3_, 14.62; *p* < 0.001; OR_grade 5 vs. grade 3_, 22.88, *p* = 0.004) and intervention performed at the day of trauma (OR 3.22; *p* = 0.014) were powerful predictors of occurrence of clinical progress.

**Conclusion:**

Our data suggest that routine CT imaging 48 h post trauma can be safely omitted for patients with low- and high-grade blunt renal injury as long as they remain clinically stable. Patients with high-grade renal injury have the highest risk for clinical progress; thus, close surveillance should be considered especially in this group.

## Background

The kidney is the most common organ within the genitourinary system to be injured during trauma and occurs in 8–10% of patients with abdominal injuries [1]. Blunt renal trauma is usually caused by sudden deceleration, leading to contusion or laceration of the renal parenchyma. Increasing recreational activities in our alpine region such as skiing and mountain biking lead to even more frequent blunt trauma accidents. With nearly 50 million tourist nights spent per year and several referring hospitals in the region, the Medical University Hospital of Innsbruck stands as a high-volume level 1 trauma centre in the central alpine region. We already shared data of our experience with blunt renal trauma in children with one of the largest cohorts known [2]. The American Association for the surgery of trauma (AAST) has implicated a radiological globally applied classification to define grades 1–5 of renal trauma [3]. In the past few years, a switch from primary surgical interventions to a more conservative approach has been generally observed [4]. Whereas low-grade trauma has always primarily been assigned to non-operative management, high-grade kidney injuries were often directly forwarded to immediate surgical intervention. In the past years, non-operative management including minimally invasive interventions such as stenting due to urinoma formation or angiographic coiling to control bleeding has become the established gold standard and treatment of choice for all haemodynamically stable patients, regardless of their primary injury grade [5–8].

Management and clinical utility of repeat routine imaging in high-grade (grade 4 and 5) renal trauma remains a very controversial issue in current literature and data are lacking. Up to now, case series in adults use CT to follow-up renal trauma, although already in 2009, ultrasonography (US) was confirmed to be an efficient alternative modality to monitor blunt trauma in a paediatric cohort [2, 9]. Whereas current EAU Guidelines on Paediatric Urology recommend a close follow-up with ultrasound 48 to 72 h after the initial CT scan in stable patients irrespective of trauma severity [10, 11], the Guidelines of the American Urological Association (AUA) and the European Association of Urology (EAU) recommend early repeat imaging in high-grade injury within two to four days after trauma to minimize the risk of missed complications [6, 12]. In patients with low-grade (grade 1 to 3) renal trauma, repeat CT imaging can be omitted as long as no symptoms occur according to current guidelines [1]. However, these recommendations are based on a retrospective series from 2010 with 138 patients undergoing conservative treatment and repeat routine imaging more than 48 h after trauma [13]. A few studies already gave first evidence that repeat CT imaging in uncomplicated also high-grade injuries is not justified [13, 14]. Data of larger cohorts to emphasize this retaining patient management is yet lacking.

In addition to the now well-established conservative management without the occurrence of any unnecessary surgical complications, potential danger of the radiation exposure must also be considered when routinely performing CT scans without an absolute indication especially in young people. Risk of carcinogenic effects of CT is assumed to be small for an individual patient [15], yet one should also review the risk in terms of the trauma patient population. Concerns about carcinogenic risks have already encouraged attempts to reduce CT imaging when not inevitably necessary [16, 17].

Our study presents the largest described case series of isolated blunt renal trauma patients with routine follow-up CT imaging 48 h post trauma. The aim of the study was to assess the diagnostic value and clinical utility with regard to intervention rates of routine repeat control CT imaging in uncomplicated patients with blunt renal trauma 2 days post trauma in comparison to CT control at the time of clinical symptoms (clinical progress).

## Materials and methods

This is an observational study based on a retrospective analysis of the level 1 trauma urological centre database. Analysis of admitted uncomplicated patients with blunt renal trauma between 2000 and 2020 was performed. Consent of the local ethics commission of the Medical University Innsbruck was obtained with the approval number 1001/2021. Research work was performed in accordance with the 1964 Helsinki declaration, its later amendments and institutional ethical standards based on good clinical practice [18].

### Patient cohort

**Fig. 1:**
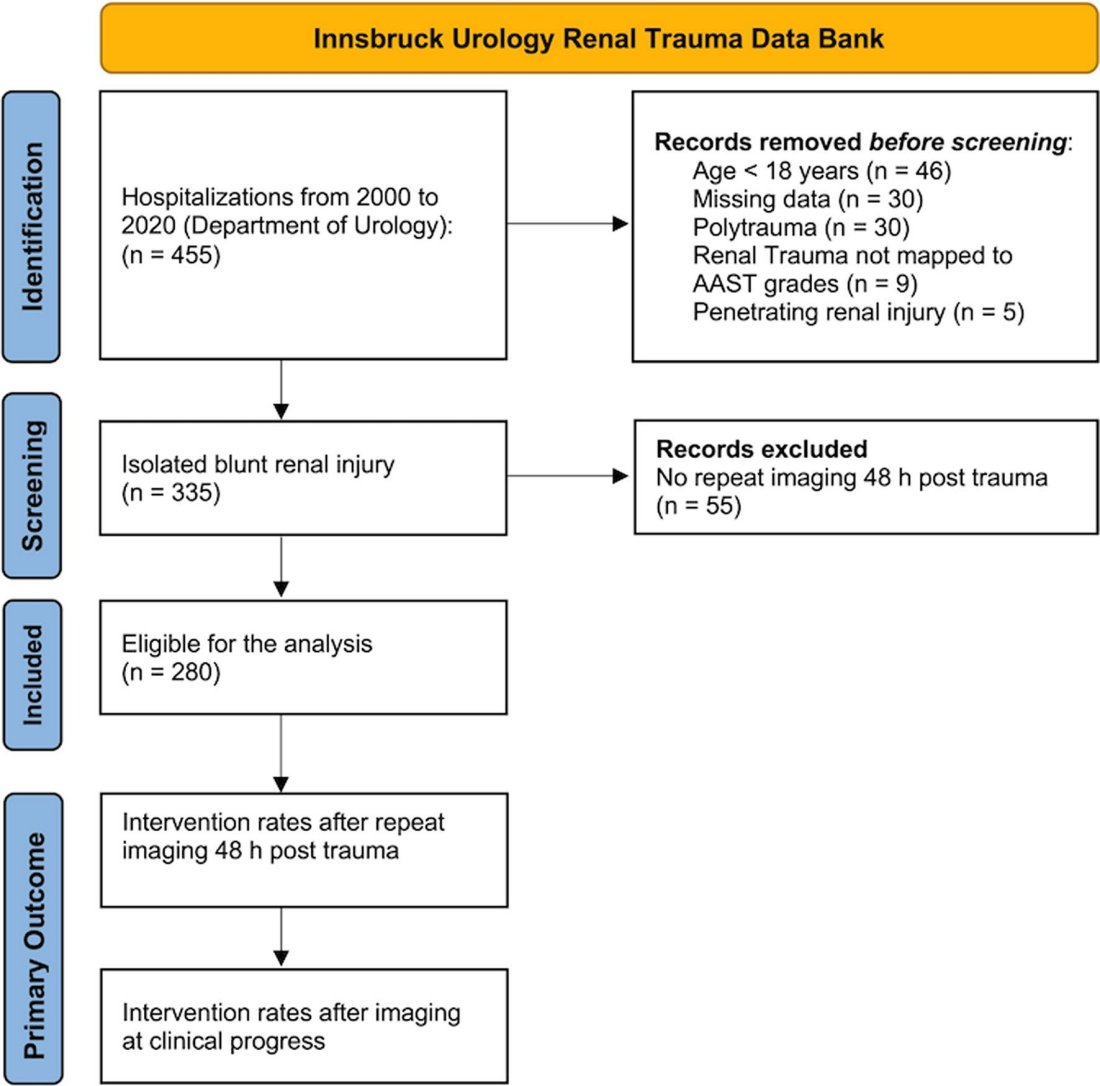
Patient flowchart

Polytrauma (i.e. high velocity car accidents, fall of great height) patients are known to have injuries of other organ systems as well. We therefore focussed on solely recreational activities, mostly leading to isolated contusion and deceleration injuries of the kidney. All patients who were admitted with diagnosis of isolated renal injury due to leisure activities (winter and summer sports) were identified via the centre’s inpatient registry. A retrospective review of all patients’ medical records and imaging results was taken. Primary endpoint was the rate of intervention after CT as repeat routine in asymptomatic patients or, if the patient developed any, at onset of clinical symptoms. Inclusion criteria were presence of isolated blunt renal trauma due to leisure activities, age > 18 years and repeat imaging 48 h post trauma. Penetrating kidney trauma, polytraumatic injuries, missing data, age < 18 years, renal trauma not mapped to AAST grading and no repeat CT imaging after 48 h were seen as exclusion criteria. A patient flow chart is presented in Fig. 1.

### Diagnostic categorization and AAST renal injury grading scale

**Fig. 2:**
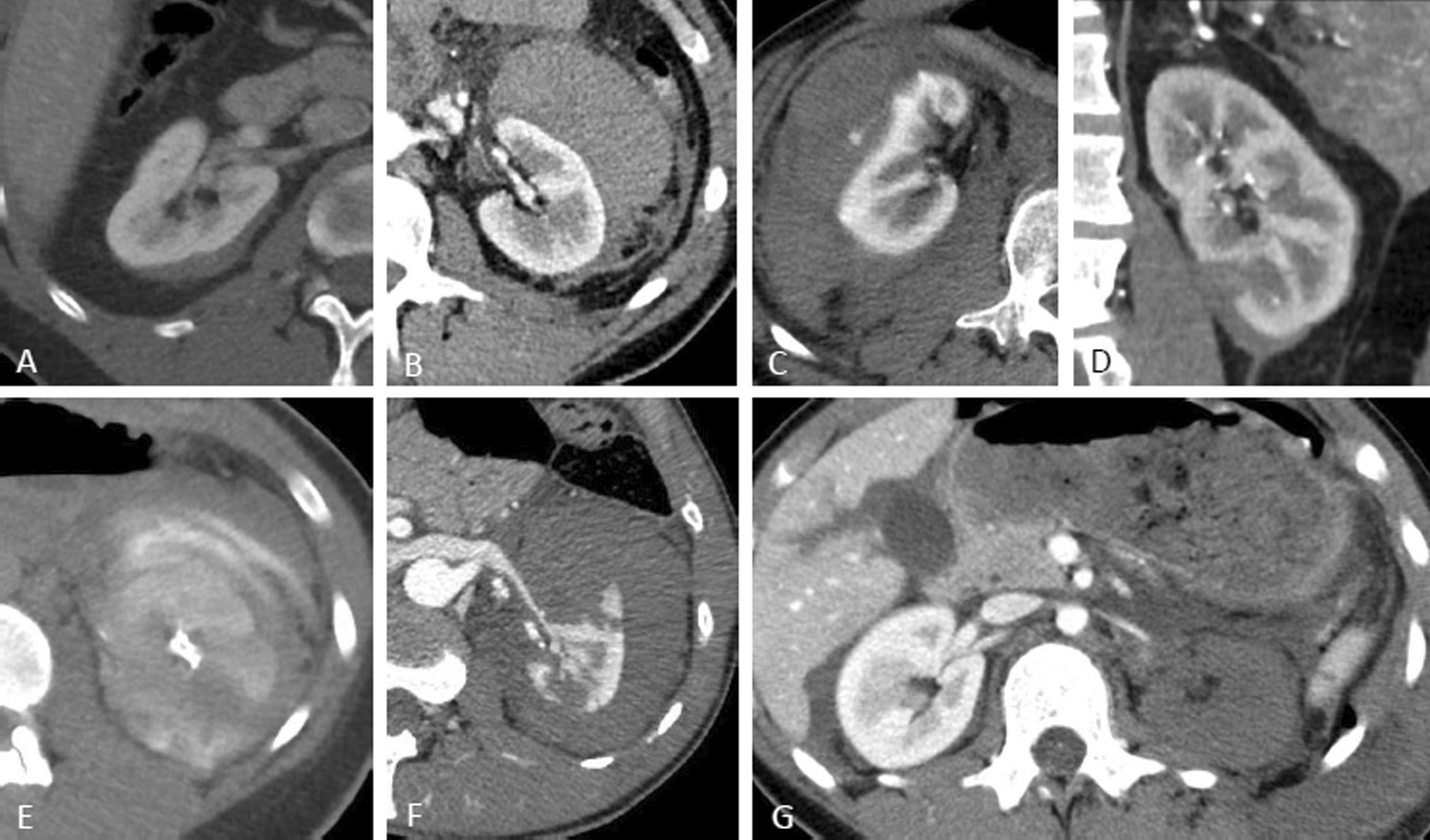
Classification of renal trauma using the American Association for the Surgery of Trauma (AAST) renal injury scale. Arterial and portal venous phase imaging is recommended for evaluation. Clinical or imaging findings suggesting collecting system injury should be followed by a delayed excretory phase to detect urine extravasation. The imaging classification criteria are as follows: grade 1 (**A**): subcapsular haematoma or contusion, without laceration; grade 2 (**B–C**): superficial laceration ≤ 1 cm depth not involving the collecting system with no evidence of urine extravasation (**B**) or perirenal haematoma confined within the perirenal fascia (**C**); grade 3 (**D**): laceration > 1 cm not involving the collecting system, vascular injury or active bleeding confined within the perirenal fascia; grade 4 (**E**): laceration involving the collecting system with urinary extravasation, laceration of the renal pelvis, vascular injury to segmental renal artery or vein, segmental infarctions without associated active bleeding or active bleeding extending beyond the perirenal fascia, grade 5 (**F–G**): shattered kidney (**F**), avulsion of renal hilum or laceration of the main renal artery or vein or devascularized kidney (**G**) [19]

Descriptive patient evaluation at admission included demographic data, inpatient stay, laboratory results (haematocrit, haemoglobin, electrolytes, liver enzymes, and baseline creatinine, urea), urinalysis, evaluation of macrohaematuria, grading in primary CT imaging, as well as intervention date and method. Renal trauma was diagnosed using CT at admission. CT included at least a portal venous and excretory phase and an arterial phase. Injuries were classified according to the 2018 revised version of the AAST [19], as seen in Fig. 2. Injuries were categorized into low-grade (grade 1–3) and high-grade renal trauma (grade 4 and 5). All patients were observed during hospitalization, including bed rest, fluids, prophylactic antibiotics, analgetics and regular laboratory controls and monitoring. We performed a CT scan after 48 h in all inpatients based on our institutional practice. Additionally, a CT was always indicated when acute clinical symptoms (haematuria, fever, flank pain, haemodynamic instability) occurred to assess for acute complications and the need for subsequent intervention. With reference to the current standard of management of renal trauma, a non-surgical observational therapy approach was primarily chosen whenever possible. Acute intervention was performed in patients with haemodynamic instability, grade 5 vascular injury and life-threatening circumstances. Furthermore, within these subgroups we correlated rate and extent of intervention comparing the decision on either the base of the 48 h repeat CT versus onset of clinical symptoms. Minimally invasive therapy included angiography with selective angioembolization and ureteral JJ stent/drainage. Open surgery included renal exploration with reconstruction and/or nephrectomy.

### Statistical analysis

Variables were summarized as counts (*n*) and percentages (%) for categorical variables and means and standard deviations (SD) for continuous variables. Baseline and outcome characteristics were tabulated stratified by injury severity, low-grade (grade 1–3) versus high-grade (grade 4–5). Differences between groups were evaluated using Fisher’s exact tests for categorical variables and independent t-tests for quantitative variables. A multivariate logistic regression model was used to estimate adjusted odds ratios (ORs) and 95% confidence intervals (CIs) to identify predictive factors for clinical progress. All statistical tests were two-sided at a significance level of 0.05. SPSS, version 26.0 (IBM Corp., Armonk, NY, USA), was used for statistical analysis.

## Results

Two-hundred and eighty patients were admitted at our department due to isolated blunt renal trauma with repeat CT imaging after 48 h from 2000 to 2020. The mean (range) age was 38.1 (18–78) years. Of these, 87.1% (*n* = 244) were male. Renal trauma was classified as follow: 7 (2.5%) grade 1, 50 (17.9%) grade 2, 93 (33.2%) grade 3, 119 (42.5%) grade 4 and 11 (3.9%) grade 5. Thus, 150 (53.6%) patients were classified as low-grade trauma and 130 (46.4%) as high-grade injury.

### Baseline characteristics

Mean (range) time of inpatient stay was 8.5 (3–30) days, with a statistically significant longer stay for high-grade trauma patients (mean 10.6 days) than in low-grade trauma (mean 6.6 days, *p* < 0.001). On time of admission, mean baseline haemoglobin level on trauma day was 12.1 g/dl, with a significant difference between the low-grade (13.2 g/dl) and high-grade trauma (11.0 g/dl, *p* < 0.001) group. Baseline creatinine levels at trauma day were 0.98 mg/dl in low-grade and 1.05 mg/dl in high-grade traumata (*p* = 0.021). Analysis showed no differences concerning presence of macrohaematuria at trauma between the two groups, thus being not predictive for high-grade renal injury. Moreover, gross haematuria at trauma was not an independent prognostic factor for clinical progress in the further course (odds ratio [OR] 2.32; *p* = 0.127). None of the patients needed haemofiltration at any time and none of the patients died. Clinical progress was—unsurprisingly—significantly more frequent in high-grade renal injuries, 2.0% (n = 3) in low-grade and 24.6% (*n* = 32) in high-grade injuries (*p* < 0.001). Of important note is that clinical progress after trauma occurred after a mean (± SD) period of 5.3 (± 4.1) days with no significant difference between both groups (low-grade vs. high-grade: 6.7 vs. 5.2; *p* = 0.561).

### Intervention rates

Overall intervention rate at any time was 36.1% (*n* = 101) with a significant higher rate in high-grade compared to low-grade injuries (72.3% vs. 4.7%; *p* < 0.001), respectively. Focusing on the day of trauma, 59 (21.1%) patients with high-grade trauma needed immediate interventions. Of these 81.4% (*n* = 48) could be managed via minimally invasive surgery (ureteral stenting in 30 patients and selective angioembolization in 18 patients), the remaining 18.6% (*n* = 11) underwent open surgery, of which nine (81.8%) grade 5 injuries required immediate nephrectomy. Overall nephrectomy rate was 3.9% (*n* = 11), including also 2 patients at the time of clinical progress. All patients with nephrectomy were classified as grade 5 vascular renal trauma.

In only 5.7% (*n* = 16) cases, intervention was performed based on repeat CT imaging 48 h after trauma. Among these, five were (3.3%) low-grade and 11 (8.5%) high-grade injuries (*p* = 0.075). Most patients (73.7%) were treated with minimally invasive procedures including ureteral JJ stent due to expanding urinoma (*n* = 8) and selective angioembolization (*n* = 3) due to pseudo-aneurysm. In four patients, we decided to perform open renal reconstruction to due expanding urinoma based on renal pelvis rupture. Patient history confirmed existing ureteropelvic junction (UPJ) obstruction in all 4 patients.

On the contrary, intervention rate due to clinical symptoms or laboratory alterations was higher with 12.5% (*n* = 35) and onset of clinical symptoms, such as fever, macrohaematuria, haemodynamic instability or flank pain was on average 5.3 days post injury. In detail, whereas all low-grade trauma patients (*n* = 3) received minimally invasive therapy, 12 (37.5%) of 32 high-grade trauma patients were referred to open surgery. Although the rate of open surgery in high-grade trauma was similar between CT control 48 h post trauma (36.4%) and at clinical progress (37.5%), nephrectomy rate was higher at clinical progress (16.6% vs. 0%), Table 1.

**Table 1:** Descriptive patient characteristics of the study population

Characteristics	Total (*N* = 280)	Grade 1–3 injury (*N* = 150)	Grade 4–5 injury (*N* = 130)	*p* Value*
Injury grade, *n* (%)				–
1	7 (2.5%)	7	–	
2	50 (17.9%)	50	–	
3	93 (33.2%)	93	–	
4	119 (42.5%)	–	119	
5	11 (3.9%)	–	11	
Age [years], mean (SD)	38.1 (18.0)	38.1 (16.9)	38.2 (19.3)	0.945
Male sex, *n* (%)	244 (87.1%)	129 (86.0%)	115 (88.5%)	0.594
Inpatient stay [days], mean (SD)	8.5 (5.1)	6.6 (3.9)	10.6 (5.4)	< 0.001
Intervention (overall), *n* (%)	101 (36.1%)	7 (4.7%)	94 (72.3%)	< 0.001
Intervention (trauma day), *n* (%)	59 (21.1%)	0 (0.0%)	59 (45.4%)	< 0.001
Severity of intervention (trauma day)				–
Minimal-invasive	48 (81.4%)	–	48 (81.4%)	
Open	11 (18.6%)	–	11 (18.6%)	
Clinical progress	35 (12.5%)	3 (2.0%)	32 (24.6%)	< 0.001
Severity of intervention (clinical progress)				0.536
Minimal-invasive	23 (65.7%)	3 (100.0%)	20 (62.5%)	
Open	12 (34.3%)	0 (0.0%)	12 (37.5%)	
Time of clinical progress after trauma [days], mean (SD)	5.3 (4.1)	6.7 (3.5)	5.2 (4.2)	0.561
Intervention (CT control 48 h), n (%)	16 (5.7%)	5 (3.3%)	11 (8.5%)	0.075
Severity of intervention (CT 48 h)				0.516
Minimal-invasive	11 (73.3%)	4 (100.0%)	7 (63.6%)	
Open	4 (26.7%)	0 (0.0%)	4 (36.4%)	
Creatinine [mg/dl] at trauma day, mean (SD)	1.01 (0.22)	0.98 (0.21)	1.05 (0.24)	0.021
Hb [mg/dl] at trauma day, mean (SD)	121.4 (23.3)	131.6 (17.6)	109.6 (23.6)	< 0.001
Macrohaematuria at trauma, *n* (%)	148 (52.9%)	75 (50.0%)	73 (56.2%)	0.338

^*^p Values from Fisher’s exact test for categorical variables, and independent t-tests for quantitative variables

In a multivariate logistic regression model, grade 4 trauma (OR_grade 4 vs. grade 3_, 14.62; 95% confidence interval [CI]: 4.16–51.38; *p* < 0.001), grade 5 renal trauma (OR_grade 5 vs. grade 3_, 22.88; 95% CI: 2.73–191.54; *p* = 0.004) and intervention at trauma day (OR, 3.22; 95% CI: 1.27–8.15; *p* = 0.014) were found to be independent predictive factors for clinical progress, Table 2.

**Table 2:** Multivariate logistic regression analysis to predict clinical progress

Baseline factors	Odds ratio (OR)	95% confidence interval (95% CI)	*p* Value*
Age (per one-year increase)	1.001	0.980–1.022	0.934
Sex (male vs. female)	0.851	0.246–2.951	0.800
Gross haematuria (yes vs. no)	0.510	0.214–1.212	0.127
Severity of trauma		–	0.001
Grade 4 (vs. Grade 3)	14.624	4.162–51.384	< 0.001
Grade 5 (vs. Grade 3)	22.878	2.733–191.536	0.004
Intervention at trauma day (yes vs. no)	3.219	1.271–8.149	0.014

Multivariate model adjusted for age, sex, haematuria at baseline, severity of trauma and intervention at trauma day. CI, confidence interval; OR, odds ratio

## Discussion

To our best knowledge, we present the largest cohort with routine CT imaging 48 h after blunt renal trauma in uncomplicated patients. Collision and deceleration trauma mechanisms often result in isolated laceration and contusion of the kidney, with 47% (*N* = 150) of trauma resulting in high-grade renal traumata in our cohort. Of these, 45.4% required immediate intervention, 81.4% of which were minimal-invasive interventions and 18.6% of which required open surgery at the day of trauma. In the past decades, there has been a paradigm shift concerning the management of high-grade renal trauma from immediate open surgical exploration to minimally invasive management. However, no prospective randomized controlled trial or systemic review compared effectiveness of conservative and surgical management in high-grade renal trauma; thus, level of evidence is low [20]. Nevertheless, there are only a few absolute indications for immediate surgical intervention, the majority can be successfully managed non-operatively [21]. In our cohort, the rate of immediate intervention at trauma day was 21.1% affecting only high-grade renal injuries. The primary AAST classification additionally has been shown to be predictive for the need of surgical intervention [22, 23] and there is mounting evidence that standardized routine re-imaging in the absence of clinical symptoms or changed laboratory findings has little impact on decision-making regarding intervention or clinical outcome [24] even in high-grade injuries [25].

Concerning follow-up, the SIU guidelines recommend repeat CT 36–72 h after a grade 4 injury with damage to the collecting system (Grade C, SIU), [26]. The AUA guidelines also recommend follow-up imaging for high-grade injuries at 48 h (Grade C, AUA) [5] and the EAU guidelines on day two to four after trauma in high-grade injuries [1]. The rationale for repeat imaging until today is early identification and treatment of complications. Literature has already proposed a solely selective approach to reimaging for even high-grade blunt renal trauma only in patients with clinical symptoms [14]. It was also shown that intensive reimaging of grade 3 and 4 renal injuries did not alter clinical decision making [25].

In our cohort, overall intervention rate based on repeated CT imaging in asymptomatic patients was very low with 5.7% and independent of the severity of renal trauma (low-grade 3.3% vs. high-grade 8.5%). In most patients (73.3%), minimally invasive management was sufficient. Moreover, 80% of patients were treated due to expanding urinoma. One can of course argue here that based on the asymptomatic status a conservative therapeutic strategy would also have been possible, decreasing the intervention rate at 48 h to 1.1% (*n* = 3 with angioembolization due to pseudo-aneurysm). In line with our results, a French multicentric study including 927 patients presented that systemic repeated imaging following renal trauma changed the therapeutic management in only 5.1% of asymptomatic patients [27]. We therefore conclude that routine imaging also for high-grade renal injuries in the absence of any clinical symptoms or clinical findings is likely unnecessary.

Onset of clinical symptoms was on average seen on day five to six post trauma. Concluding, this would mean that after routine CT after 48 h or even, as recommended by the EAU [1, 6], AUA [5] and SIU [26], after the latest four days, the patient would need another obligate CT with accompanying radiation dosage when acute symptoms occur. Two studies with a cohort size of up to 218 patients reported on selection of repeat CT imaging guided not by trauma grading but rather based on the presence of urine extravasation in addition to clinical and laboratory criteria [28] and have shown that selective reimaging of renal injuries based on clinical and laboratory criteria would have detected all complications [24]. In our study, intervention after imaging due to onset of clinical symptoms was observed in 12.5% of patients and had a higher rate of open surgery (37.5%) and nephrectomy (16.6%), leading to the conclusion that patients who really had a clinical need for a CT were the ones who received it. Two studies also strengthen our argument by already showing that routine reimaging after 48 h without a clinical indication was narrowly beneficial and contributed to change the treatment in less than 1%, proposing to omit repeat imaging in the absence of symptoms [13, 14]. Loftus et al*.* stated that individuals with grade IV trauma who received routine CT follow-up imaging were more likely to undergo an operation in the absence of symptoms and received more radiation during their hospital stay. Additionally, in line with our data, reimaging was not associated with an increase in urological complications [29]. By selective choice in the use of CT, one would not only contribute to a health system cost reduction, but also to a reduction in radiation exposure when considering long-term risks associated with CT in trauma imaging in young patients [30, 31]. There is a proposal for haemodynamically stable but symptomatic patients to use ultrasound (US) for first evaluation and to proceed to a CT only when US remains inadequate [24], based on the fact that significant urinomas can be sufficiently detected by US as already shown in a children cohort [9]. Here it is of notice that US achieves better diagnostics in children due to their physical constitution, yet it may be used as method of choice also in follow-up evaluation in adult patients with renal injuries.

Several limitations must be mentioned. The major limitation of our study includes the retrospective, single-centre study design, which limits statistical power. In addition, no control group (blunt renal trauma without repeat CT imaging at 48 h) was included, so we have no head-to-head comparison. Our findings, however, imply that routine CT re-imaging at 48 h can be safely omitted not only in uncomplicated low-grade, but also in high-grade renal trauma patients.

In conclusion, our cohort demonstrates that high-grade renal trauma and presence of intervention on the day of trauma are independent factors in predicting clinical progression. Importantly, macrohaematuria at baseline did not correlate with the grade of injury and was not an important predictive factor of further clinical progression. It is yet important to note that patients with high-grade blunt injury remain at a high risk for clinical progress; thus, close surveillance including lab tests and sonography is recommended in this subgroup. We therefore propose that clinical symptoms (fever, flank pain, haemodynamic instability) and laboratory results, such as decreasing haemoglobin or elevating creatinine should guide the decision to perform repeat CT imaging. Based on our data, we conclude that current recommended repeat CT imaging does not predict clinically necessary interventions and can be omitted in both low- and high-grade patients without clinical symptoms indicative of worsening conditions.

## Data Availability

The data that support the findings of this study are available from the database of our institution, responsibility for the integrity of the data and the accuracy of the data is taken by the corresponding author.

## Abbreviations

AAST: American Association of the Surgery of Trauma
AUA: American Urological Association
CT: Computer tomography
EAU: European Association of Urology
US: Ultrasound
